# A Pyridazine-Containing Phthalonitrile Resin for Heat-Resistant and Flame-Retardant Polymer Materials

**DOI:** 10.3390/polym14194144

**Published:** 2022-10-03

**Authors:** Minjie Wu, Kaixiong Yang, Yuanyuan Li, Jianxin Rong, Dianqiu Jia, Zhiyi Jia, Kimiyoshi Naito, Xiaoyan Yu, Qingxin Zhang

**Affiliations:** 1 Hebei Key Laboratory of Functional Polymers, School of Chemical Engineering and Technology, Hebei University of Technology, Tianjin 300401, China; 2 Polymer Matrix Hybrid Composite Mat Grp, National Institute for Materials Science (NIMS), 1-2-1 Sengen, Tsukuba 305-0047, Japan; 3 Tianjin Key Laboratory of Materials Laminating Fabrication and Interface Control Technology, Hebei University of Technology, Tianjin 300401, China

**Keywords:** phthalonitrile, pyridazine ring, low melting point, high heat-resistant, flame-retardant

## Abstract

In this study, a novel phthalonitrile monomer containing a pyridazine ring, 3,6-bis[3-(3,4-dicyanophenoxy)phenoxy]pyridazine (BCPD) with a low melting point (74 °C) and wide processing window (178 °C), was prepared by a nucleophilic substitution reaction. The molecular structure of the BCPD monomer was identified by Fourier transform infrared spectroscopy (FTIR), and nuclear magnetic resonance spectroscopy (NMR). Poly(BCPD) resins were derived from the formulations by curing at 350 and 370 °C. The thermoset that was post-cured at 370 °C demonstrated outstanding high heat-resistant (glass transition temperature (*T*_g_) > 400 °C, 5% weight loss temperature (*T*_5%_) = 501 °C, *Y*_c_ at 900 °C > 74%) and was flame-retardant (limiting oxygen index (LOI) = 48)). Further, the poly(BCPD) resin simultaneously exhibited a superior storage modulus, which could reach up to 3.8 Gpa at room temperature. Excellent processability and heat resistance were found for phthalonitrile thermosets containing the pyridazine ring, indicating poly(BCPD) resin could be potentially applied as high-temperature structural composite matrices.

## 1. Introduction

Phthalonitrile resins have excellent comprehensive properties and can be widely used as engineering plastics in the areas of electronics, automobile manufacturing, military industry, and other fields. Currently, various resins with different structures have been developed to improve the properties and expand their application fields. In the initial reports of phthalonitrile resins, most of the structures of the synthesized monomers are based on benzene rings [1,2]. 

To obtain phthalonitrile resins with high thermal stability and better mechanical properties, various rigid aromatic ring structures were introduced into the monomer molecular chain [3,4,5,6]. However, with the improvement of thermal stabilities and mechanical properties of the phthalonitrile resins, the phthalonitrile monomer still has a high melting point (*T*_m_) and narrow processing window, which seriously hinders their practical production and applications [7]. To improve the processability of phthalonitrile resins, the molecular design of phthalonitrile monomers was carried out. For example, compounds containing flexible groups were used as raw materials to prepare new monomers [8,9,10,11,12]. Oligomeric aromatic ether phthalonitrile monomers with different degrees of polymerization were prepared by controlling the feed ratio [7,13,14,15,16,17]. However, the oligomerized phthalonitrile monomers have a relatively low cyano group density, which weakens the curing degree of the resulting polymers. A number of flexible groups may damage the mechanical properties of the phthalonitrile resins [7,16,18,19].

In our previous study, a phthalonitrile monomer containing pyrimidine structure, 4,6-bis[3-(3,4-dicya-nophenoxy)phenoxy]pyrimidine (BCPM), was synthesized [20]. The analysis results show that the addition of the pyrimidine structure significantly enhances the performance of phthalonitrile resin. The introduction of nitrogen heterocycles into phthalonitrile is conducive to improving the performance of the resin. Therefore, in this study, pyridazine was incorporated into the molecular structure of the phthalonitrile resin. Resorcinol and 3,6-dichloropyridazine were employed as the raw materials to synthesize a phthalonitrile monomer containing ether bonds and pyridazine ring, 3,6-bis[3-(3,4-dicyanophenoxy)phenoxy]pyridazine (BCPD). It was found that the BCPD monomer with the pyridazine ring exhibited a lower melting point than those of reported monomers and the introduction of the pyridazine ring further promoted the mechanical properties and heat resistance of phthalonitrile resin.

## 2. Materials and Methods

### 2.1. Materials

3,6-Dichloropyridazine (99.0%) was provided by Shanghai Chemical Technology Co., Ltd. (Shanghai, China). Resorcinol (99.0%) was purchased from Yinuokai Technology Co., Ltd. (Beijing, China). Toluene (99.0%), and anhydrous potassium carbonate (K_2_CO_3_, 99.0%) was obtained from Creus Fine Chemical Co., Ltd. (Tianjin, China). 4-Nitrophthalonitrile (98.0%) was supplied by Yuanye Biological Technology Co., Ltd. (Shanghai, China). 4-(aminophenoxy)phthalonitrile (APPH) was prepared by our laboratory. *N*,*N*-dimethylformamide (DMF, 99.5%) was obtained and purchased from Hengshan Chemical Technology Co., Ltd. (Tianjin, China). 

### 2.2. Synthesis of BCPD Monomer

Resorcinol (13.21 g, 0.12 mol), 3,6-dichloropyridazine (8.94 g, 0.06 mol), and anhydrous potassium carbonate (24.87 g, 0.18 mol) were first put in a three-necked flask, and then 75 mL of N,N-dimethylformamide (DMF) and 15 mL of toluene were added to the above mixture. The reaction was kept at 150 °C under a nitrogen atmosphere, and water as a by-product was removed by azeotrope with toluene. After 10 h of reaction, vacuum distillation was performed at 60 °C to remove the toluene. 4-Nitrophthalonitrile (20.78 g, 0.12 mol) was added to the mixture and the temperature was raised to 90 °C for 8 h. Finally, the reaction system was cooled to room temperature, and the reactant was poured slowly into deionized water. The precipitate was filtered and washed by deionized water. The crude product was purified by recrystallization for characterization (yield: 86%).

### 2.3. Preparation of Poly(BCPD) Resins

The BCPD monomer and APPH (10:1) were poured into a pre-prepared aluminum mold. The mixture was degassed in a vacuum oven at 200 °C. After degassing was completed, the compound was thermally cured by heating in an oven. Poly(BCPD)-x was finally obtained, and this x represents the last temperature segment of the curing procedure. The curing procedure for poly(BCPD)-350 was as follows: 190 °C/2 h, 220 °C/2 h, 250 °C/4 h, 280 °C/3 h, 310 °C/4 h, and 350 °C/4 h. The curing procedure for poly(BCPD)-370 was as follows: 190 °C/2 h, 220 °C/2 h, 250 °C/4 h, 280 °C/3 h, 310 °C/4 h, 350 °C/4 h, and 370 °C/4 h.

### 2.4. Characterizations

Infrared measurements were performed on a Bruker Vector 22 Fourier transform infrared (FTIR) spectrometer (Billerica, MA, USA) to obtain FTIR spectra of the BCPD monomer and polymers. ^1^H NMR (400 MHz) and ^13^C NMR (100 MHz) spectra were performed on a Bruker AV400 nuclear magnetic resonance spectrometer (NMR) using deuterated dimethyl sulfoxide (DMSO-d6) as the solvent. The wide-angle X-ray diffraction (WAXD) was recorded on a MiniFlex600 (Shimadzu Corporation, Kyoto, Japan) with Nifiltered Cu K_α_ radiation (λ = 0.15406 nm) at 40 kV and 15 mA. Differential scanning calorimetric (DSC) analysis was collected on a TA Q20 (Waltham, MA, USA) at various heating rates with a nitrogen flow rate of 50 mL/min. The thermogravimetric analysis (TGA) data for the polymers were measured by a TA Instruments Q600 (Waltham, MA, USA) at a heating rate of 10 °C/min from 50 to 1000 °C under an inert or air atmosphere, separately. Dynamic mechanical analysis (DMA) was performed by a DMA Q800 (TA Instruments) (Waltham, MA, USA) scanning with a heating rate of 5 °C/min and a frequency of 1 Hz in a single cantilever mode.

## 3. Results

### 3.1. Characterization of BCPD Monomer

**Scheme 1:**
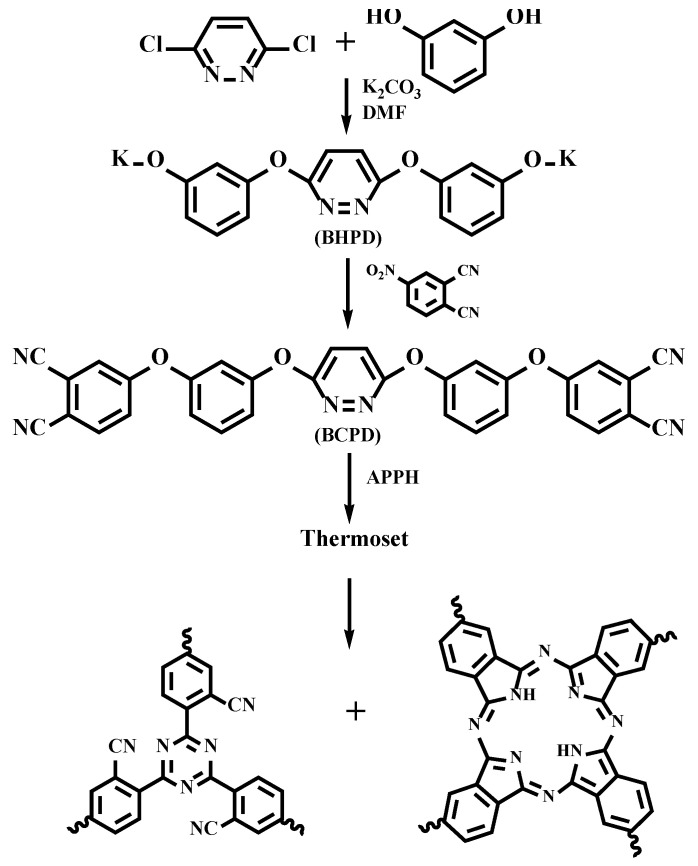
The preparation process of BCPD monomer and polymer.

3,6-Dichloropyridazine reacted with resorcinol in DMF solvent to form potassium salt intermediate in the presence of K_2_CO_3_, and then 4-nitrophthalonitrile was added to the reaction solution to obtain a BCPD monomer (Scheme 1).

**Figure 1:**
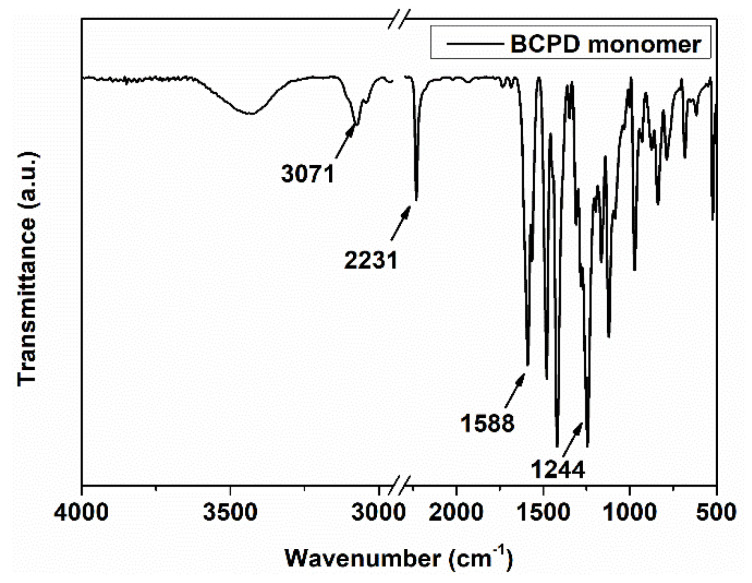
FT-IR spectrum of BCPD monomer.

The molecular structure of the monomer was characterized by FITR and NMR. The FTIR spectrum of the BCPD monomer is shown in Figure 1. The stretching vibration absorption peak of the C–H bond in aromatic rings appeared at 3071 cm^−1^. The absorptions at 2231 and 1244 cm^−1^ were assigned to the stretching vibration of the cyano group (–CN) and ether bond (C–O–C), respectively. The absorption at 1588 cm^−1^ was attributed to the stretching vibration of C=N in the pyridazine ring [21]. The FTIR results can initially prove the successful synthesis of the BCPD monomer.

**Figure 2:**
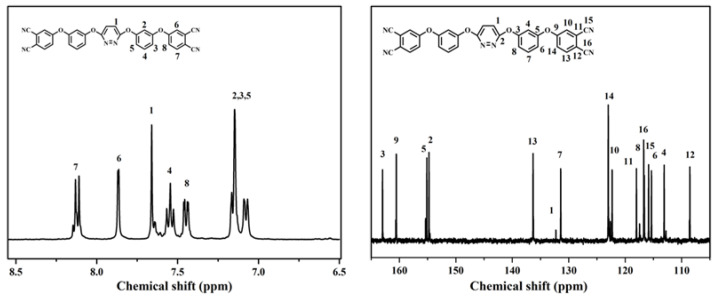
^1^H NMR (**left**) and ^13^C NMR (**right**) spectra of the BCPD monomer.

**Figure 3:**
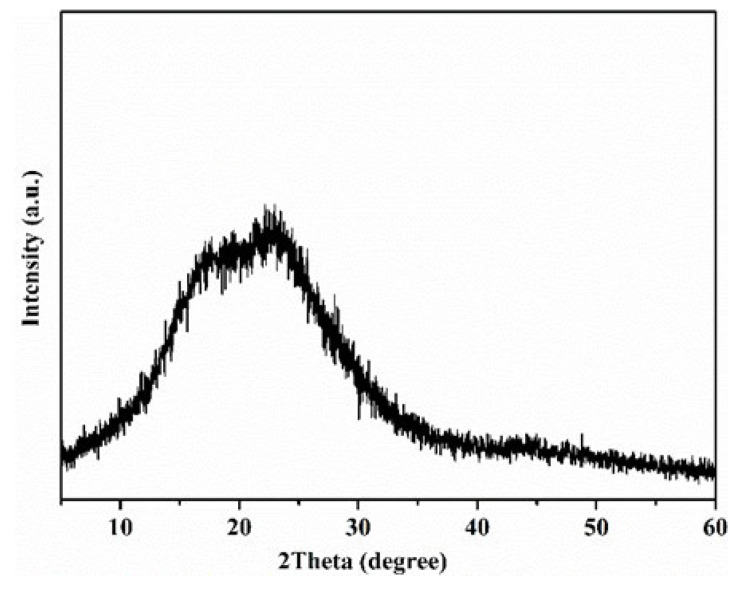
WAXD curve of the BCPD monomer.

NMR analysis was performed to further analyze the structure of the BCPD monomer, and the results were displayed in Figure 2. In the ^1^H NMR spectrum, the two doublets (d) at 8.12 and 7.87 ppm, and the doublet of doublets (dd) at 7.45 ppm belonged to the protons on the phenyl rings containing cyano groups. The singlet (s) at 7.66 ppm was assigned to the protons on the pyridazine ring. The multiple peaks in the range of 7.25–6.97 ppm was assigned to the protons on the phenyl rings with two ether bonds. In the ^13^C NMR spectrum, the peaks at 154.74 and 132.40 ppm corresponded to the carbon atoms in the pyridazine ring. The signals at 116.71 and 115.84 ppm were attributed to the carbon atoms of the cyano groups, and the other peaks were attributed to the carbons on the phenyl rings. The NMR data also indicated the successful preparation of the monomer. The WAXD patterns of the BCPD monomer are shown in Figure 3. It can be found that the BCPD monomer only presented a non-crystalline hump at 2θ = 22.8° without other sharp crystalline peaks.

### 3.2. Curing Behaviors

**Figure 4:**
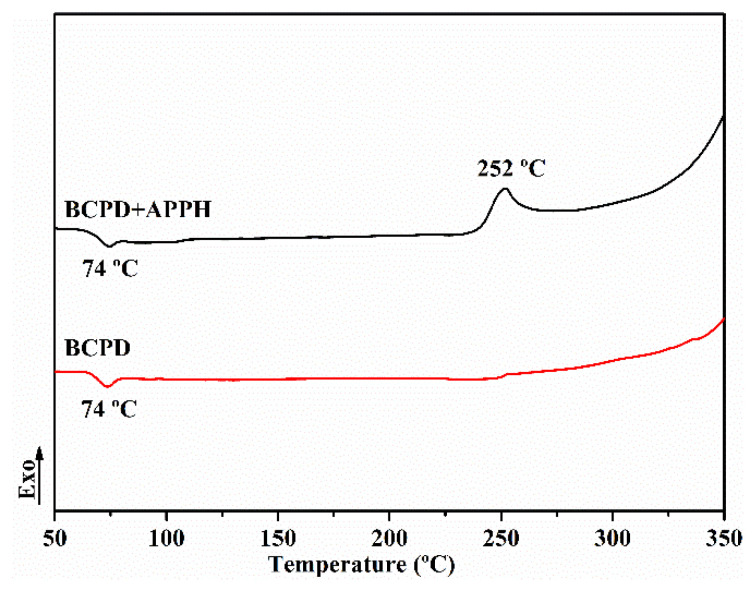
DSC curves of BCPD monomer and BCPD/APPH mixture.

The processing performance of the BCPD monomer was analyzed by DSC [3,9]. Figure 4 exhibited the DSC curves. The monomer DSC curve only exhibited an obvious endothermic peak at 74 °C, corresponding to the *T*_m_ of the monomer. The absence of an exothermic peak indicates that the monomer had not undergone polymerization, and the addition of a curing agent was necessary for catalyzing the reaction of the monomer. In the DSC curve of the BCPD/APPH mixture, a strong exothermic peak can be found at 252 °C, besides the melting peak of the monomer, indicating the occurrence of a curing reaction. It can be concluded that the BCPD monomer possessed a wide processing window of approximately 178 °C. Presumably, the electronegativity of the N atoms in pyridazine was greater than that of its adjacent C atoms, resulting in a decrease in the electron density of C atoms and an increase in the bond length of carbon-oxygen bonds. Therefore, the molecules rotated more freely.

It is worth noting that although the molecular structure of BCPD and BCPM monomer is similar, the *T*_m_ of BCPD monomer is much lower than that of the BCPM monomer in our previous study. An analogous phenomenon also appears in other reported monomers [11,22]. However, one of the differences between BCPD and BCPM monomer is that the former is non-crystalline, while the latter is crystalline. A comparison of the *T*_m_ for several monomers is presented in Table 1. 

**Figure 5:**
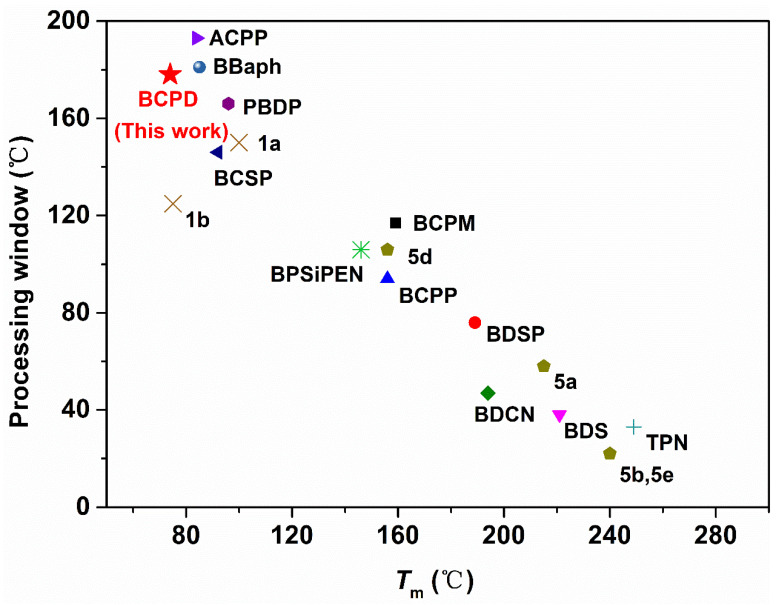
The processing performance of phthalonitrile in this work is compared with that reported in the literature [3,4,11,12,20,21,22,23,24,25,26,27,28].

It is suggested that monomers with low *T*_m_ are usually amorphous [11,23,25], while monomers with high *T*_m_ are usually crystalline [4,20,22,23]. In previous studies, increasing the flexibility of molecular segments is the main strategy for reducing the *T*_m_. Based on this phenomenon, the hypothesis of lowering the melting point by reducing the crystallization of monomers was proposed. Moreover, the DSC curves suggested satisfying processability (*T*_m_: 74 °C; processing window:178 °C), which is obviously superior to previously reported phthalonitrile resins. In order to show the excellent processing performance of BCPD monomer more intuitively, a comparison of *T*_m_ and the processing window of BCPD with other phthalonitrile is shown in Figure 5 [3,4,11,12,20,21,22,23,24,25,26,27,28]. Hence, predictably, the research results greatly expand on the development of phthalonitrile resin in composite materials.

**Figure 6:**
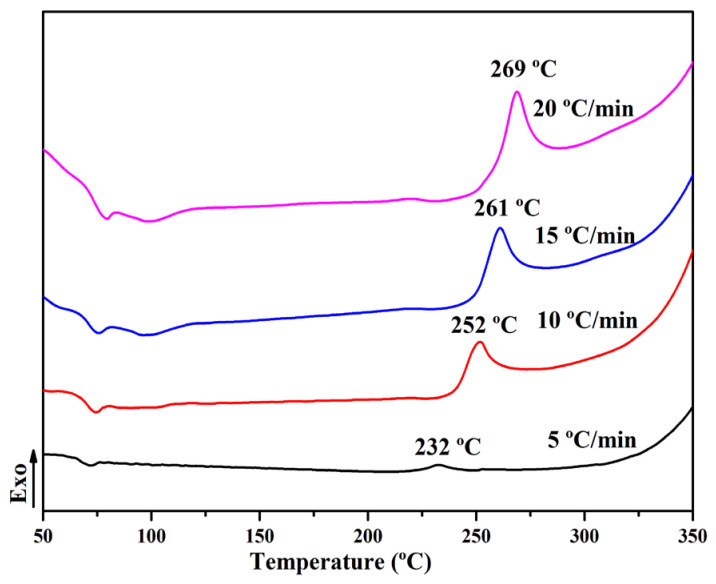
The DSC curves of BCPD/10 wt.% APPH at different heating rates.

Using the non-isothermal method, the mixture of the BCPM monomer with 10 wt.% APPH was tested by DSC at respective heating rates of 5, 10, 15, and 20 °C/min. The results are shown in Figure 6. Exothermic peaks at heating rates of 5, 10, 15, and 20 °C/min were observed at 232, 252, 261, and 269 °C, respectively. It can be seen that increasing the heating rate will cause the curing peak to shift to a high temperature. When the temperature rise is slow, the monomer is heated more thoroughly, and the curing reaction is carried out at a lower temperature [29]. 

**Figure 7:**
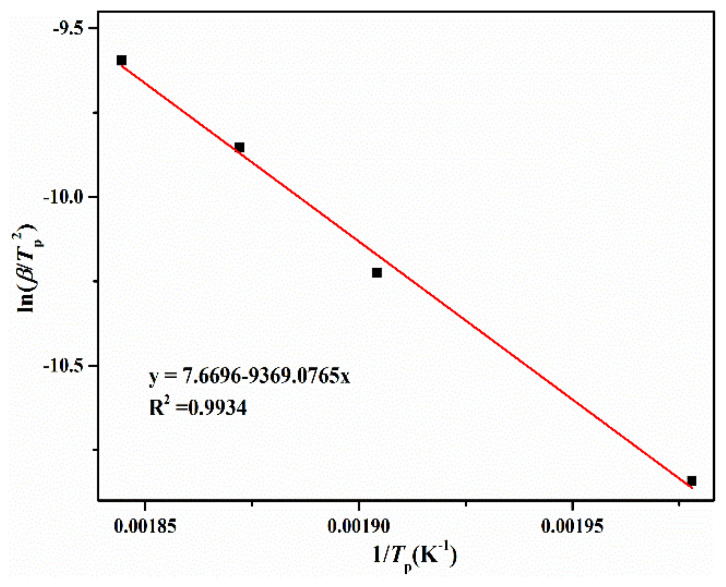
The relationship between ln(β/$T_{p}^{2}$) and 1/*T*_p_.

The apparent activation energy (*E*_α_) can intuitively reflect the difficulty of the polymerization reaction. The curing reaction is easy to proceed with when the *E*_α_ is low. It can be calculated according to the Kissinger Equation (1) [30]:

(1) $$-ln\frac{\beta }{T_{p}^{2}}=\frac{E_{\alpha }}{RT_{p}}-ln\frac{A^{\prime }}{RE_{\alpha }}$$

where β represents the heating rate (°C/min) of DSC, *T*_p_ is the temperature (K) when the exothermic peak reaches the peak, and R is the ideal gas constant (8.314 J·mol^−1^·K^−1^). The kinetic parameters of the curing reaction were computed according to the DSC test results, which are listed in Table 2. Then linear fitting was performed on ln(β/$T_{p}^{2}$) and 1/*T*, as shown in Figure 7. Finally, the *E*_α_ was calculated as 77.89 kJ/mol, which is similar to those of reported thermoset resins in literatures [31,32,33].

### 3.3. Structural Characterization of Poly(BCPD) Resin

**Figure 8:**
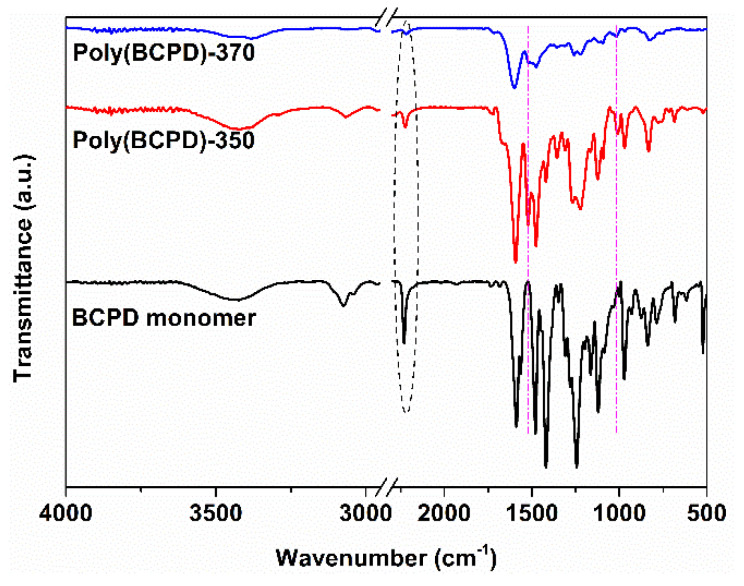
FT-IR spectra of BCPD monomer and poly(BCPD) resins.

The FTIR spectra of the BCPD monomer and polymers were compared to explore the curing process. As shown in Figure 8, in the FTIR spectra of poly(BCPD)-350 and poly(BCPD)-370, the intensity of the absorption peak for cyano groups was significantly weakened, compared to that of the BCPD monomer. The new infrared absorption peaks corresponding to the triazine ring and phthalocyanine ring structure were found at 1522 and 1012 cm^−1^, respectively [34,35]. It is implied that the curing reaction of the BCPD monomer was successfully catalyzed by the curing agent APPH. At the same time, no obvious infrared absorption peaks for the isoindoline structure were observed in the FTIR spectra of the polymers, indicating that the structures of triazine rings and phthalocyanine rings were mainly formed after curing. During the curing process, the formation of cross-linked structures restricts the relative movement between molecules. The probability of collision between cyano groups decreases, which eventually results in some unreacted cyano groups remaining inside the resin. Therefore, a weak cyano absorption peak is always observed in the FTIR spectra of the polymers.

**Figure 9:**
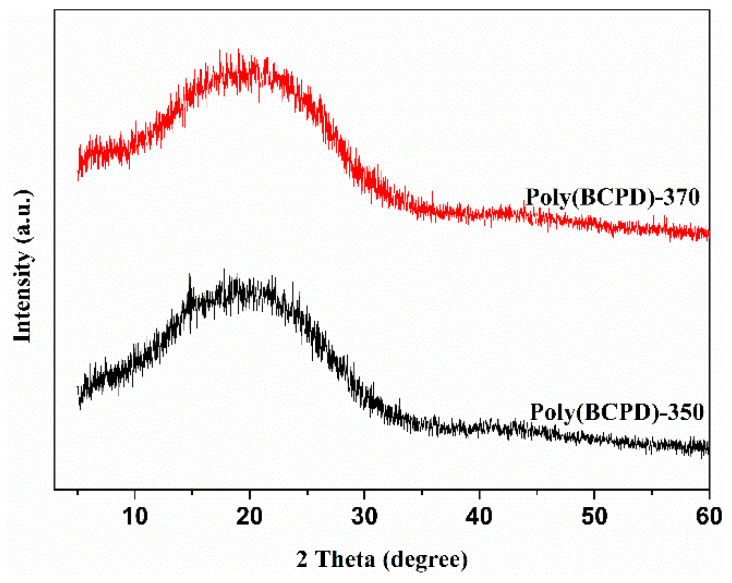
WAXD curves of the poly(BCPD) resins.

The WAXD patterns of the poly(BCPD) resins are shown in Figure 9. Poly(BCPD)-350 and poly(BCPD)-370 also exhibit non-crystalline humps with diffraction angles of 2θ = 19.4° and 2θ = 19.7°, respectively. According to Equation (2), the average interatomic distance of polymers was calculated [35,36]:

(2) $$R=\frac{5}{4}\times \frac{\lambda }{2sin\theta }=1.25d_{Bragg}$$

where λ represents the wavelength of X-rays and 2θ represents the diffraction angle. The average atomic distances of poly(BCPD)-350 and poly(BCPD)-370 were calculated to be 0.50 and 0.49 nm, respectively, by substituting the amorphous diffraction peak of the polymer into the formula. The decrease in the interatomic distance indicates that the cross-linking reaction was more sufficient. Therefore, the curing temperature and time can be appropriately increased to obtain a resin with better performance.

### 3.4. Thermal and Thermal-Oxidative Stabilities

**Figure 10:**
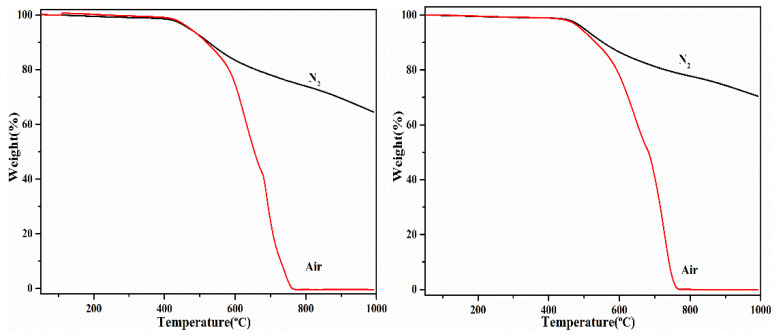
TGA curves of the polymer A (**left**) and polymer B (**right**) under atmosphere of nitrogen and air.

The TGA tests were tested under two atmospheres to characterize the thermal stability of polymers. The TGA curves of polymer A and polymer B are shown in Figure 10 and the specific thermal performance parameters are listed in Table 3. 

Under the nitrogen (N_2_) atmosphere, the poly(BCPD)-350 and poly(BCPD)-370 showed 5% mass degradation (*T*_5%_) at 470 and 501 °C, and the carbon yield (CR) at 900 °C was 70% and 74%, respectively. In the air atmosphere, the thermal weight loss of poly(BCPD)-350 and poly(BCPD)-370 reached 5% at 472 and 492 °C, respectively. The cross-linked network structure composed of nitrogen heterocycles is the main reason why phthalonitrile resins have outstanding heat resistance. Compared with poly(BCPD)-350, poly(BCPD)-370 shows better thermal performance, also verifying that the cross-linked structure was improved by increasing the reaction temperature and extending the reaction time [11].

The flame-retardant performance of BCPD polymer can be calculated by the Van Krevelen Equation (3) [3,37], which is shown as follows:

(3) $$LOI=17.5+0.4\times {CR}^{\prime }$$

The limiting oxygen index ((*LOI*) is the minimum volume fraction of oxygen when the polymer can burn in a mixture of oxygen and nitrogen. It can be used to characterize the flame-retardant property of the material. CR is the char residue of the polymer at 850 °C in nitrogen. The CR of poly(BCPD)-370 at 850 °C is 76.2%, and the *LOI* value is 48 by calculation. It is generally considered that a polymer material is a flame-retardant material when its LOI value exceeds 26 [38]. Hence, poly(BCPD) resin can be regarded as a candidate for flame-retardant material with excellent performance.

### 3.5. Dynamic Mechanical Analysis

**Figure 11:**
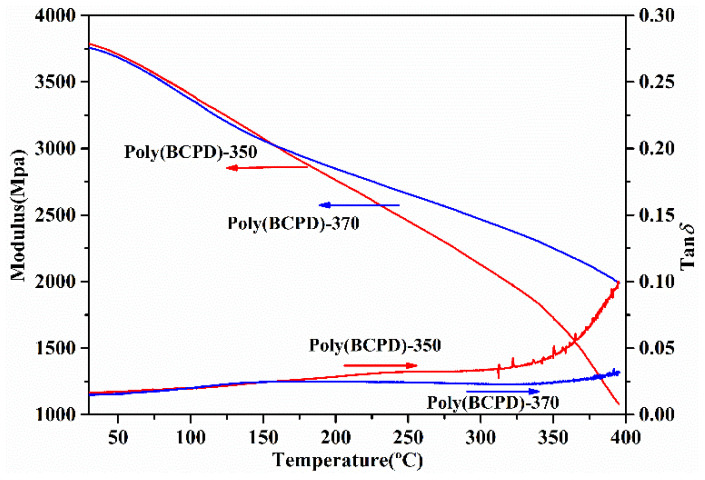
DMA curves of the poly(BCPD) resins.

As a high-performance engineering plastic, phthalonitrile resins often have superior thermo-mechanical properties. In this research, the thermo-mechanical property of BCPD polymers was investigated by DMA. According to Figure 11, both poly(BCPD)-350 and poly(BCPD)-370 have outstanding mechanical behavior. The storage modulus of polymers at 30 °C is 3781 and 3756 MPa, respectively. Although the storage modulus gradually weakens with increasing temperature, and polymer B could reach 1989 MPa at 400 °C. The storage modulus of poly(BCPD)-370 at 400 °C is higher than those of most previously reported phthalonitrile resins [7,18,39,40,41,42,43]. Meanwhile, there is no obvious peak in the loss factor curves of the two polymers, implying that the glass transition temperature (*T*_g_) of the polymers exceeds 400 °C. The pyridazine structure has greater rigidity and stronger polarity. It can interact with the nitrogen heterocycle in the cross-linked structure to hinder the movement of molecular segments and improve mechanical properties.

### 3.6. Water Uptake

**Figure 12:**
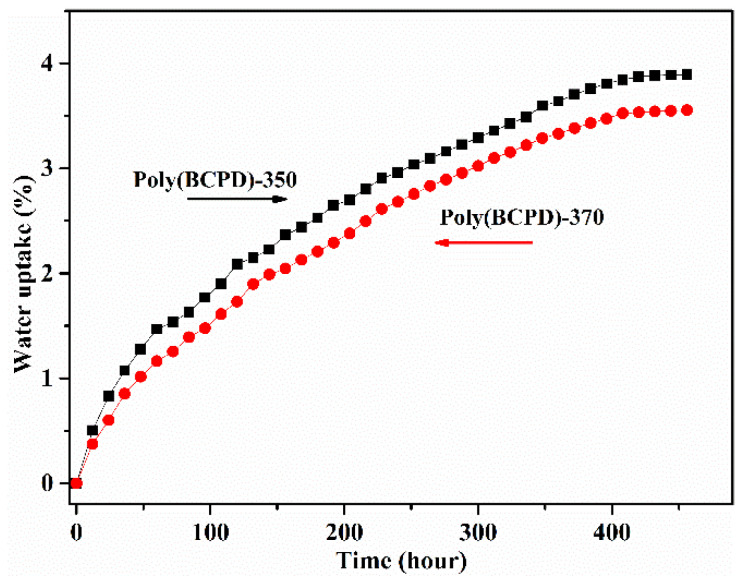
Water uptake curves the poly(BCPD) resins.

Poly(BCPD)-350 and poly(BCPD)-370 were immersed in deionized water and weighed at 12 h intervals until the sample mass tended to be constant. The water uptake was obtained according to the Equation (4) as follows:

(4) $$Water\;uptake\;\left(\%\right)=\frac{M_{2}-M_{1}}{M_{1}}\times 100\%$$

where *M*_1_ is the initial mass of the poly(BCPD) resins and *M*_2_ is the mass of the polymers after soaking. The curves of water uptake of poly(BCPD)-350 and poly(BCPD)-370 with time is shown in Figure 12. In the early stage of the experiment, the water uptake increased rapidly. With the increase of the soaking time, the water uptake increased slowly and eventually reached the maximum value of 3.98 wt.% and 3.57 wt.%, respectively. The poly(BCPD) resins with dense cross-network structure exhibit excellent water resistance and can be used in a humid environment, expanding their application range. The degree of crosslinking of the polymer was enhanced with an increasing curing temperature and time. Since it is more difficult for water molecules to penetrate into the crosslinking polymer, the water uptake of poly(BCPD)-370 was lower than that of poly(BCPD)-350, which is consistent with the TGA and DMA test results.

## 4. Conclusions

In the study, a novel phthalonitrile monomer BCPD with a pyridazine ring was successfully synthesized through a nucleophilic substitution reaction. The monomer could be melted at 74 °C, and the processing window was as wide as 178 °C. The poly(BCPD)-370 presents outstanding thermal stability and thermomechanical properties. Its initial decomposition temperature (*T*_5%_) was 501 °C and the glass transition temperature (*T*_g_) was over 400 °C. Moreover, the LOI was as high as 48. The poly(BCPD) resin exhibited low water uptake, showing application prospects in a highly humid environment. The melting point of the amorphous BCPD monomer was lower than that of the crystalline BCPM monomer which we previously reported. The incorporation of a pyridazine ring into the phthalonitrile further promotes the thermomechanical properties and heat resistance of phthalonitrile resin. Considering all the advantages above, the poly(BCPD) resin would be a suitable and potential candidate for heat-resistant and flame-retardant polymer materials.

## Figures and Tables

**Table 1:** The comparison of several phthalonitriles’ *T*_m_.

Sample	*T*_m_ (°C)	Crystalline or Amorphous	Ref
BDCN	194	crystalline	[4]
BCPM	159	crystalline	[20]
BDSP	189	crystalline	[22]
BCPP	156	crystalline	[21]
BDS	221	crystalline	[23]
BCSP	92	amorphous	[11]
ACPP	84	amorphous	[24]
PBDP	96	amorphous	[25]
BCPD	74	amorphous	This work

**Table 2:** ln(β/$T_{p}^{2}$), lnβ and 1/*T*_p_ at different heating rates.

*β/*(°C/min^−1^)	*T*_p_/(K)	ln(*β*/$T_{p}^{2}$)	1/*T*_p_/(K^−1^)
5	494.65	−10.84	1.98 × 10^3^
10	509.75	−10.22	1.90 × 10^3^
15	520.25	−9.85	1.87 × 10^3^
20	528.25	−9.60	1.84 × 10^3^

**Table 3:** Thermal performance parameters of the poly(BCPD) resins in nitrogen and air atmosphere.

Sample	In Nitrogen			In Air		
	*T*_5%_/°C	*T*_10%_/°C	*CR*/%	*T*_5%_/°C	*T*_10%_/°C	*CR*/%
Polymer A	470	525	64.4	472	518	0
Polymer B	501	553	70.4	492	533	0

## Data Availability

Not applicable.
